# A Model-Driven Method for Pylon Reconstruction from Oblique UAV Images

**DOI:** 10.3390/s20030824

**Published:** 2020-02-04

**Authors:** Wei Huang, San Jiang, Wanshou Jiang

**Affiliations:** 1 State Key Laboratory of Information Engineering in Surveying, Mapping and Remote Sensing, Wuhan University, 129 Luoyu Road, Wuhan 430079, China; hw1006@whu.edu.cn; 2 School of Computer Science, China University of Geosciences, Wuhan 430074, China; jiangsan@cug.edu.cn; 3 Collaborative Innovation Center of Geospatial Technology, Wuhan University, 129 Luoyu Road, Wuhan 430079, China

**Keywords:** pylon reconstruction, unmanned aerial vehicle images, inner distance shape context, Markov Chain Monte Carlo

## Abstract

Pylons play an important role in the safe operation of power transmission grids. Directly reconstructing pylons from UAV images is still a great challenge due to problems of weak texture, hollow-carved structure, and self-occlusion. This paper presents an automatic model-driven method for pylon reconstruction from oblique UAV images. The pylons are reconstructed with the aid of the 3D parametric model library, which is represented by connected key points based on symmetry and coplanarity. First, an efficient pylon detection method is applied to detect the pylons in the proposed region, which are obtained by clustering the line segment intersection points. Second, the pylon model library is designed to assist in pylon reconstruction. In the predefined pylon model library, a pylon is divided into two parts: pylon body and pylon head. Before pylon reconstruction, the pylon type is identified by the inner distance shape context (IDSC) algorithm, which matches the shape contours of pylon extracted from UAV images and the projected pylon model. With the a priori shape and coplanar constraint, the line segments on pylon body are matched and the pylon body is modeled by fitting four principle legs and four side planes. Then a Markov Chain Monte Carlo (MCMC) sampler is used to estimate the parameters of the pylon head by computing the maximum probability between the projected model and the extracted line segments in images. Experimental results on several UAV image datasets show that the proposed method is a feasible way of automatically reconstructing the pylon.

## 1. Introduction

Modern society has become increasingly reliant on the electricity supply. Electric power systems, which provide electricity to modern society, are indispensable components of the industrial world. Pylons are the elementary facility and play a vital role in the safe and reliable operation of electric power systems. Regular inspections and monitoring the status of pylons are an important approach to ensure the safety of electric power systems. Accurate parameters and detailed types of pylon can be obtained through the reconstruction procedure. In addition, the pylon model can be used in many contexts, such as transmission corridor visualization, simulation analysis, disaster prevention, etc. 

There are seven fundamental types of data that can be used in high-voltage transmission line inspections [1]. Among those, ALS data and unmanned aerial vehicle (UAV) images are the most common data sources for 3D reconstruction of high-voltage transmission corridors and power facilities. ALS is an active remote sensing technique that can quickly obtain dense 3D point clouds with high precision [2]. It can be applied in the fields of 3D reconstruction of pylons, detailed high-voltage transmission corridor mapping, monitoring of power lines and their surroundings, etc. With a laser scanner mounted on an aircraft scanning along the high-voltage transmission corridors, 3D dense point clouds on power lines, pylons and their surroundings can be accurately measured. However, this method has some limitations that restrict its wide application. The high cost of laser scanning equipment increases the price of such inspections. In addition, due to its weight and large size, it is not convenient for regular inspections. In contrast to ALS data, UAV images can be easily captured using a UAV equipped with cameras, which is cheaper and more convenient for regular inspections [3]. The extrinsic and intrinsic parameters of cameras can be determined using the method of structure from motion (SfM) with the auxiliary information GNSS/IMU (Global Navigation Satellite System/Inertial Measurement Unit) [4,5,6]. The dense point clouds of high-voltage transmission corridors can be generated by the multi-view stereo (MVS) method. However, because of the complex structures of pylons and the lack of feature points, there are only several matched points of pylons, making pylon reconstruction from UAV images more difficult than from ALS data. 

**Figure 1:**
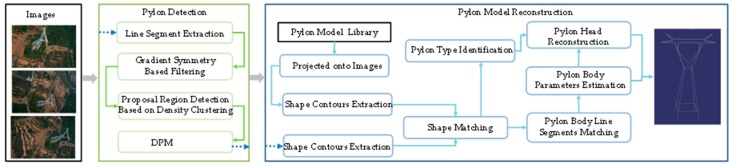
The workflow of proposed method.

The proposed method uses oblique UAV images to reconstruct the pylons. As the line segments are the most salient feature of pylon in images, the presented method tries to use the line segments to recover the 3D information of pylons instead of points. The line segments can be extracted by any line segment detector such as LSD (Line Segment Detector) [7] or EDL [8]. The workflow of the proposed method is shown in Figure 1. First, an improved pylon detection approach is proposed to detect the pylon from the UAV images efficiently. The proposed regions are computed with the consideration of gradient symmetry and the density of intersection points, and the DMP method is applied to detect the pylon in the proposed regions, which improves the efficiency of pylon detection. Second, the shape contours of pylon are extracted with alpha shape algorithm using the clustered intersection points of line segment. The prior information of the pylon is adopted to translate, rotate and project the pylon model onto each visible image. After extracting the shape contours of the projected models, the IDSC [9] method is adopted to match the two shape contours. The pylon types can be identified according to the matching cost. Third, the line segments of the pylon body are matched with the shape prior and coplanar constraint. Then, the pylon body is modeled by fitting four principle legs and four side planes with the matched line segments on pylon body. Finally, a MCMC sampler is designed to estimate the parameters of pylon head by computing the similarity between the line segments of projected pylon head model and the extracted line segments of the pylon in the images.

In this paper, a novel automatic pylon reconstruction framework from oblique UAV images is introduced. The main contributions of the proposed framework are in three aspects: (1) an efficient pylon detection method is employed to detect pylons in images, which detect the pylon in the proposed regions by considering the structural features of pylons instead of searching the whole image region; (2) the pylon body is reconstructed with the aid of a model library. The pylon model library is used to identify the pylon type and guide the initial 3D line segment matching using the IDSC method. The a priori shape and coplanar constraint of pylon body are taken into consideration to match the 3D line segments on pylon body and fit the four side planes; (3) an MCMC sampler is applied to estimate the parameters of pylon head by computing the maximum likelihood probability between the projected model and the extracted 2D line segments in images.

## 2. Related Work

Recently, there have been several high-quality algorithms focusing on 3D object reconstruction. However, according to our knowledge, the automatic image-based reconstruction of pylon has rarely been reported, and only a few works research automatic pylon reconstruction based on ALS data [10,11,12,13]. Li et al. first proposed a model-driven method to reconstruct the pylon [10]. The pylon is divided into three parts: legs, body and head. The legs and body are reconstructed by analyzing the geometry features on point clouds and the head type is classified by a SVM (Support Vector Machine) classifier. However, this method can only confirm the head type, and cannot estimate precise parameters of pylon head, and the reconstructed pylon model restricts its wide application in electric power systems, such as windage yaw simulation. Guo et al. developed a stochastic geometry method to reconstruct the pylon. The pylon points are automatically extracted from dense point clouds and an efficient global optimization function is conducted to reconstruct the pylon [11]. Zhou et al. introduced a heuristic pylon reconstruction method [13]. The pylon body is reconstructed by a data-driven strategy, and the pylon head is efficiently reconstructed by a model-driven strategy using a Metropolis-Hastings sampler coupled with a simulated annealing algorithm, which reduces the number of parameters and searching space. Chen et al. divided the pylon into three parts: inverted triangular pyramid lower structures, quadrangular frustum pyramid middle structures and complex upper structures [12]. The prior knowledge with a data-driven strategy is applied to reconstruct the lower and middle structures and the priori abstract template structures are used to reconstruct the upper structures. Similar to [13], in the proposed method, the pylon is divided into two parts: pylon body and pylon head. The reconstruction of the pylon body is based on the a priori shape and plane constraint and the pylon head parameters are estimated using the MCMC method. The four methods can efficiently and successfully reconstruct pylons, but due to the high cost and weight of laser scanning equipment, ALS data cannot satisfy the frequent inspection requirement. 

In addition to the ALS-based pylon reconstruction methods, the related image-based works on power facilities reconstruction are pylon line segment matching [14] and power line reconstruction [1,15]. Hofer et al. introduced a line segment matching method to reconstruct the wire objects (e.g., pylons) [14], but the results are only 3D line segments without semantic or topological information. When the number of images is not high enough, especially in the situation of high-voltage transmission line inspection, Hofer’s method does not usually perform well, and the matched 3D line segments cannot reconstruct the pylon. Zhang et al. focused on automatic obstacle detection within the power line corridor. An automatic power line measurement method was proposed, which acquired the spatial position of power lines based on epipolar constraint (PLAMEC) and extracted dense point clouds based on semi patch matching. Fryskowska proposed a wavelet-based method to extract the 3D power lines from noisy point clouds generated by image dense matching algorithm [15]. Jiang et al. designed an UAV-based oblique photogrammetric system to acquire images of transmission lines and conduct accuracy assessment tests to evaluate and explore the potential application for inspection of transmission lines [3]. The oblique photogrammetric system and the SfM pipeline are applied for data acquisition and image orientation in our work. Except for the power facilities reconstruction from UAV images, there are also many works on the power transmission lines inspection and transmission infrastructure monitoring [16,17,18]. Sampedro et al. proposed a supervised learning approach to detect and classify the pylon from UAV images [16]. The HOG (Histogram of Oriented Gradients) features were applied for training two MLP (multi-layer perceptron) neural networks. Jalil et al. applied the multi-modal sensors to capture the visible and infrared images and detect the faults or damaged components of the transmission infrastructure [17]. Han et al. aimed to detect the insulator faults from aerial images [18]. A manually labelled dataset was built for the newly designed convolutional network to solve the insulator faults detection problem.

A pylon is a typical man-made object with a special structure, and this building is the most representative example of a widely studied man-made object. The relevant literature of image-based automatic building modeling will be discussed. There exists a significant body of image-based approaches in the field of 3D building reconstruction. Generally, prior knowledge, such as parallelism, vanishing point, line-features and piece-wise plane, is taken into consideration to constrain the target 3D models [19,20,21,22,23,24,25]. Hoiem et al. proposed a single image-based method which creates a “pop-up” 3D model [19]. Kosecka and Zhang presented an approach for the automatic extraction of dominant rectangular structures with higher-level information from a single image [20]. Barinova et al. used the assumption of the Manhattan structure to reconstruct 3D models [21]. Conditional Random Field (CRF) models are employed to automatically infer polyline parameters and produce 3D building models. Without any explicit assumptions, Saxena et al. used a trained MRF to model the image depth cues and different parts relation from the image [22]. In their works, the reconstruction is based on a single image, while many other methods are based on multi-view images. Werner and Zisserman provided an automatic method that generates a coarse planar model and more detailed polyhedral models, such as windows and doors, are fitted with the guidance of the coarse model [23]. Dick et al. introduced a Bayesian and model-based method [24]. The a priori wall layout and the a priori parameters for each primitive are defined, which are partially learned from training data and partially set by expert architects. Finally, a MCMC machinery is employed to optimize the structure recovery and generate possible solutions. Xiao et al. presented an approach to reconstruct 3D models from street-level images [25]. The images are firstly semantically segmented by a supervised learning method. Then the major line structures are adopted to separate building into independent blocks. Finally, the facade is reconstructed by an inverse patch-based orthographic composition and structure analysis method from each block. These multi-view-based methods either rely on the dense point clouds generated by MVS or line segment features. However, neither of them can be directly applied to pylon reconstruction due to the special structures of pylon. 

The remainder of the paper is organized as follows: the brief descriptions of pylon library are introduced in Section 3; the details of efficient pylon detection method are described in Section 4; pylon type identification, pylon body reconstruction, and pylon head reconstruction are mentioned in Section 5; the details of experimental datasets and results are presented in Section 6; Section 7 discusses the influence factors of pylon reconstruction; and Section 8 provides the conclusions of the proposed method.

## 3. The 3D Pylon Model Library

The first step of pylon model reconstruction consists of specifying the 3D pylon models. The most widely used pylons in high-voltage transmission lines can be categorized into two classes according to the head part: that with a ring and that without a ring. The pylon with a ring in the head is generally applied in single circuit lines and the other is generally applied in multiple circuit lines. Due to data limitations, four typical types of pylon model are constructed: two with a ring and the other two without a ring in the head. If a new type of pylon emerges that is not contained in the model library, the new model should be added to the library to ensure successful pylon reconstruction.

**Figure 2:**
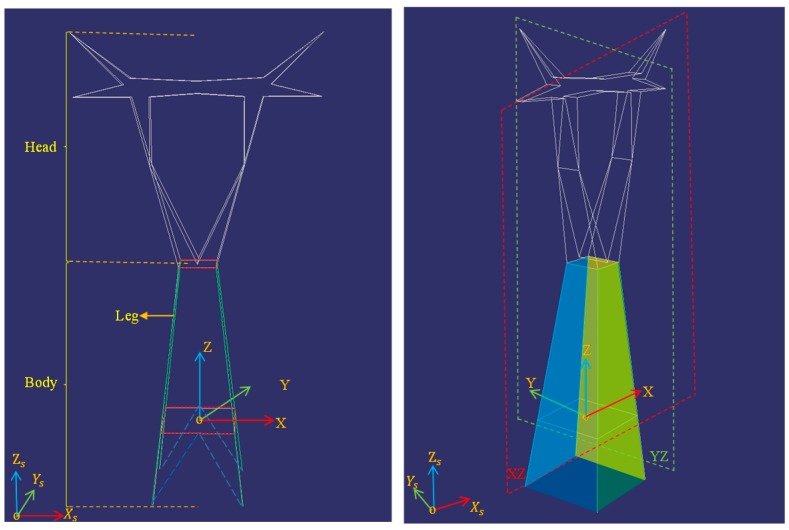
The structure of pylons. (**a**) The two divided parts of the pylon; (**b**) the four side planes and two symmetry planes of the pylon.

The pylon model is divided into two parts: the pylon body and the pylon head, and the pylon body consists of four principle legs and four side planes, as shown in Figure 2. For the four different types of pylon model, the pylon body has the same parameters and there are two horizontal rectangle structures in the body. The model library is built in full consideration of symmetry and coplanar features. For simplicity, the center of the pylon model is the origin of the right-handed coordinate system. The pylon direction is represented as the angle between the Y axis of the pylon’s local coordinate system and the $Y_{s}$ axis of the object coordinate system. The structures of pylons are mainly symmetric in the XZ plane and YZ plane. The structures on the pylon body are coplanar, with four side planes, as shown in Figure 2b.

**Figure 3:**
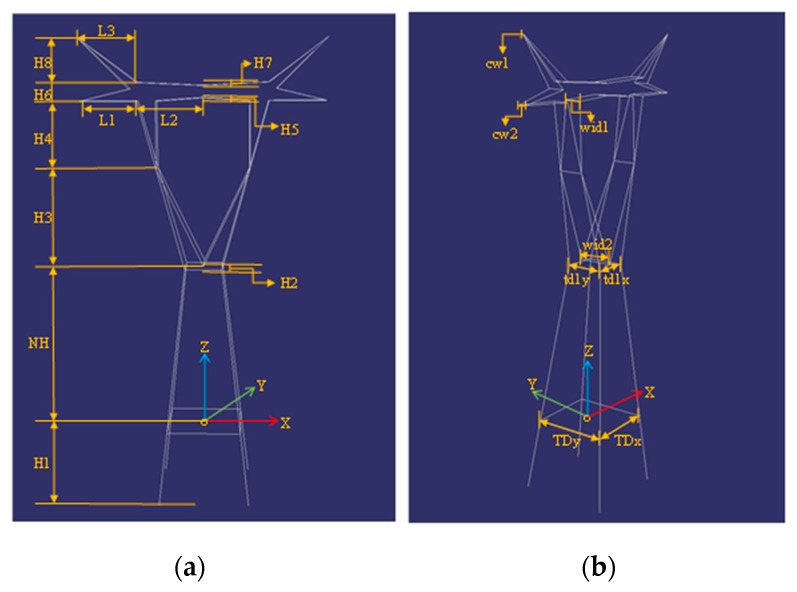

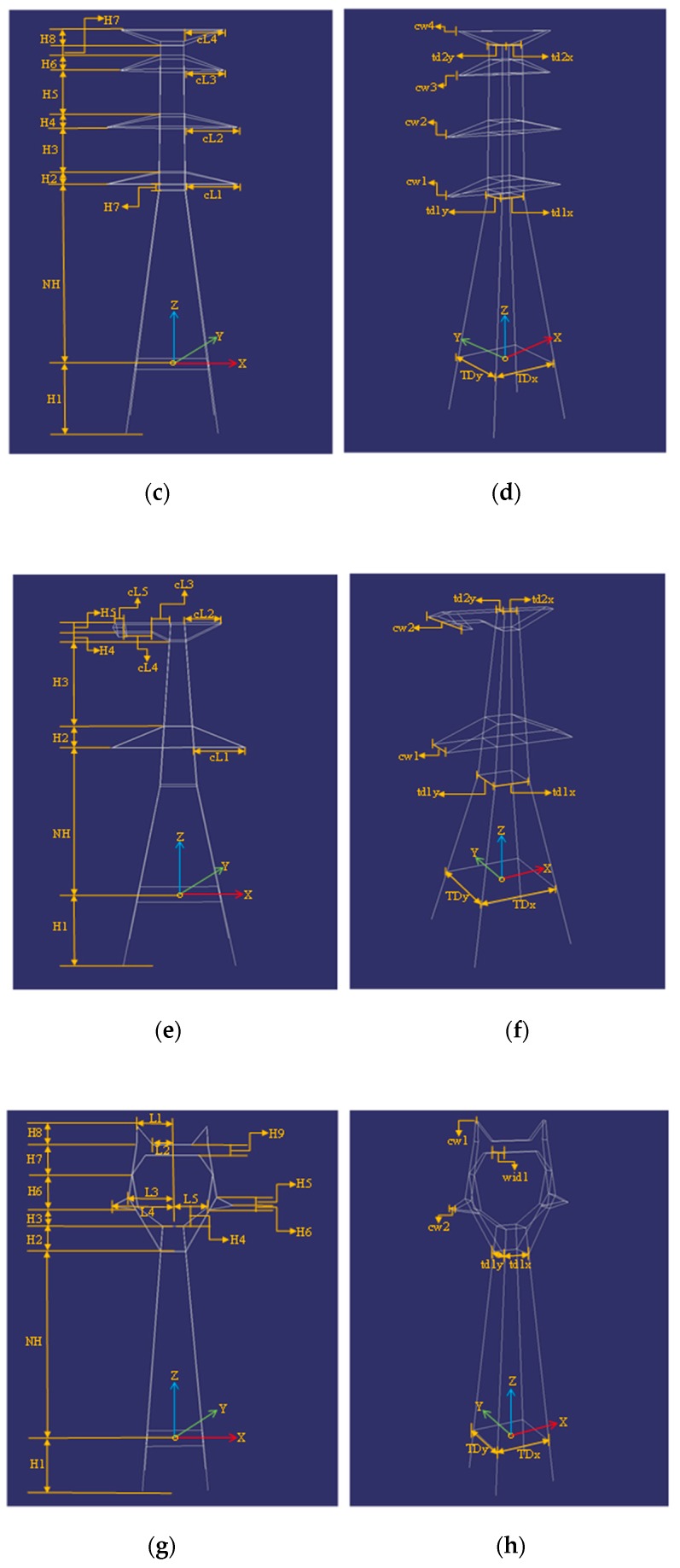
Pylon model library. (**a**,**b**) The $M_{1}$ model with different views; (**c**,**d**) the model $M_{2}$ with different views; (**e**,**f**) the $M_{3}$ model with different views; (**g**,**h**) the $M_{4}$ model with different views.

The structural parameters $p_{s}$ of the model are the series of key turning points’ distance to the XZ plane and the YZ plane of the pylon, as shown in Figure 3. In addition, each model has the common parameters $p_{c}$ of location and direction: the center point coordinates and the direction. The parameters of the four type models are defined as $\theta =(p_{s},p_{c})$. The pylon models are presented using line segments connected with turning points, which can be used to guide the line segment matching in images. In addition, each model records the four side planes’ information using the coplanar line segments.

## 4. Efficient Pylon Detection

Pylons in high-voltage transmission lines are composed of hinged steel. In the 2D image, the pylon features mesh structures and has numerous intersection points of line segment. In addition, the intersection points imply the distribution of the pylon shape contours, which can be used to find the proposed regions and extract the shape contours of the pylon. Based on these features, an efficient pylon detection method is designed. The traditional machine learning object detection method DPM (deformable part model) [26], which only needs a small amount of labeled training data while still retaining ideal results in the object detection field, is applied to perform pylon detection in this paper. This approach uses a sliding window strategy to detect objects at every location and scale in an image, which results in it having poor time efficiency and high memory consumption. Fortunately, the proposed regions can be computed by analyzing the characteristics of pylon in image. In addition, the DPM method is used only in the proposed regions to detect the pylon, which can speed up the efficiency of detection and save memory.

In this algorithm, the 2D line segments are firstly extracted using the LSD method. In addition, gradient symmetry of the line segment is employed to filter the amount of line segments from the natural background. After that, the rest of the line segments are used to calculate the valid intersection points, which can then be clustered to obtain the proposed regions. Then, the DPM method is used to detect pylons in the proposed regions.

### 4.1. Line Segment Extraction Constrained by Gradient Symmetry

The gradient symmetry of a line segment is an important feature that distinguishes man-made objects from the natural background. For natural objects, the gradient distribution of line segments is irregular. However, for man-made objects such as pylons, roads, and buildings, the gradient distribution of the line segment is commonly characterized by symmetry.

**Figure 4:**
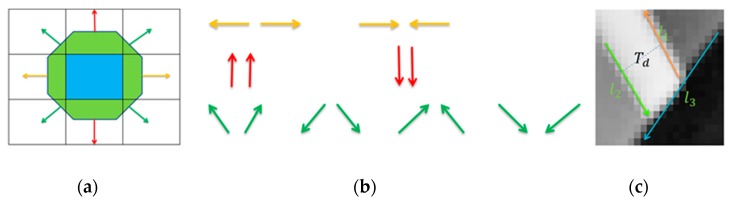
The gradient symmetry of points and line segments. (**a**) Gradient direction of a point; (**b**) the eight types of point gradient direction symmetry; (**c**) the gradient symmetry of line segments.

Katahara et al. first proposed the gradient symmetry of a point and applied it in the field of face detection [27]. The eight gradient directions and eight gradient symmetry types are defined as shown in Figure 4a,b. For a point of interest $p_{1}$, the gradient symmetry of $p_{1}$ means that there exists another point $p_{2}$ in the gradient direction of $p_{1}$ within a certain distance, and the gradient directions of $p_{1}$ and $p_{2}$ are one of the eight gradient symmetry types defined in Figure 4b. This can be extended to the gradient symmetry of a line segment: for a line segment, the gradient symmetry of the line segment means that its gradient direction is parallel to the gradient direction of its neighboring line segment at a certain distance. It can be supposed that the gradient direction of line segment is approximately vertical to the line segment direction. The problem of finding the line segments with gradient symmetry can be simplified to finding the parallel line segments within a certain distance. As shown in Figure 4c, the line segments $l_{1}$ and $l_{2}$ are extracted from image, the arrows represent the direction of the line segment, and $T_{d}$ refers to the distance between $l_{1}$ and $l_{2}$; if the directions of $l_{1}$ and $l_{2}$ are approximate parallel, these two segments can be described as having the feature of gradient symmetry within the distance $T_{d}$.

### 4.2. Pylon Proposed Region Detection Based on Density Clustering

**Figure 5:**
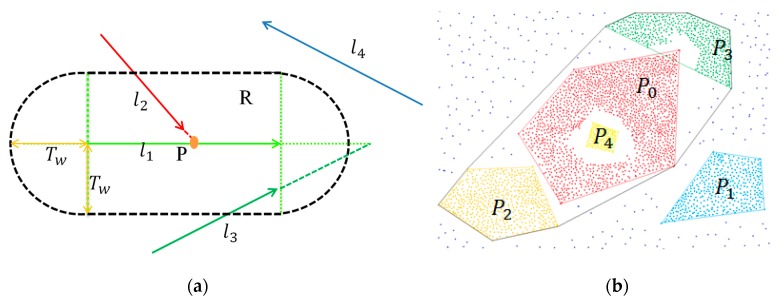
Intersection point computation (**a**) and merging of convex hulls (**b**).

After filtering an amount of background line segments, the rest of the line segments are used to compute valid intersection points. As any two non-parallel line segments will intersect with each other, the intersection point computation must be constrained to prevent too many intersection points. It is assumed that only two line segments within a certain distance will participate in intersection point computation. Furthermore, the intersection point of two line segments must not be far away from them. Based on such assumptions, a valid intersection point computation algorithm is applied, similar to that of Li and Yao [28]. With reference to Figure 5a, the buffer area R combined with a rectangular and two half of circle is designed R centers on the midpoint of $l_{1}$ and the width and height of the rectangle are $\left|l_{1}\right|$ and 2*$T_{w}$, where $\left|l_{1}\right|$ denotes the length of $l_{1}$ and $T_{w}$ is equal to the radius of the circle ($T_{w}$ is a user-defined parameter). The other line segment $l_{2}$ must have at least one endpoint in R and the intersection point must also be located in R. Then, the intersection point P is thought to be valid. 

The intersection points are distributed densely in the region of the pylon and other man-made objects. The proposed regions will be acquired after clustering these intersection points. There are many classical clustering algorithms, such as k-means, hierarchy clustering, DBSCAN [29], etc. As DBSCAN shows excellent capability to resist noise and needn’t give the number of clusters, the DBSCAN method is chosen to cluster the intersection points. In the DBSCAN method, there are two important parameters, eps and MinPts, which mean that within a radius distance eps there are at least MinPts points in the region. The two parameters should be chosen cautiously to avoid clustering noise points or segmenting the pylon into different parts. However, because of the different types of pylon and the resolution of the images, the intersection points are distributed irregularly. The two parameters cannot be chosen ideally. So the eps is conservatively set to a small value to avoid clustering points that do not belong to pylon. However, the pylon may be clustered into different parts. To solve this problem, these clusters need to be merged after the initial clustering.

An easy method is employed to merge these initial clusters. Firstly, a convex hull is calculated for each initial cluster. Then, the location correlation is computed between each convex hull and its neighbors. If the convex hull of a cluster is contained by or intersects with another cluster’s convex hull, the two clusters should be merged. Otherwise, the minimum distance between the two convex hulls is calculated. If the distance is smaller than a given threshold, the two clusters should be merged. As shown in Figure 5b, $P_{0}$, $P_{1}$, $P_{2}$, $P_{3}$, $P_{4}$ are the initial clusters. The procedure of merging the clusters is as follows:

(a) Compute the position correlation between $P_{0}$ and other clusters: $P_{4}$ is contained in $P_{0}$, $P_{0}$ intersects with $P_{3}$, $P_{0}$ does not intersect with $P_{1}$ or $P_{2}$. So the clusters of $P_{0}$, $P_{3}$ and $P_{4}$ should be merged as $P_{0}^{\prime }$.
(b) Compute the shortest distances between $P_{0}^{\prime }$, $P_{1}$ and $P_{2}$: $Dist_{\left(P_{0}^{\prime },P_{1}\right)}>T_{p}$, $Dist_{\left(P_{1},P_{2}\right)}>T_{p}$, $Dist_{\left(P_{0}^{\prime },P_{2}\right)}<T_{p}$ ($Dist_{\left(P_{i},P_{j}\right)}$ denotes the shortest distance between $P_{i}$ and $P_{j}$, $T_{p}$ is a fixed threshold), then $P_{0}^{\prime }$ and $P_{2}$ should be merged.

The number and density of the pylon’s intersection points are usually bigger than those of other man-made objects. Therefore, the point number and density threshold of the cluster can be used to filter certain clusters. The density of intersection points is calculated using Formula (1).

(1) $$T_{D}=\frac{A}{T_{Num}}$$

A means the area of the cluster’s convex hull. In addition, $T_{Num}$ means the number of the cluster’s intersection points. The rest of the clusters can be regarded as proposed regions.

**Figure 6:**
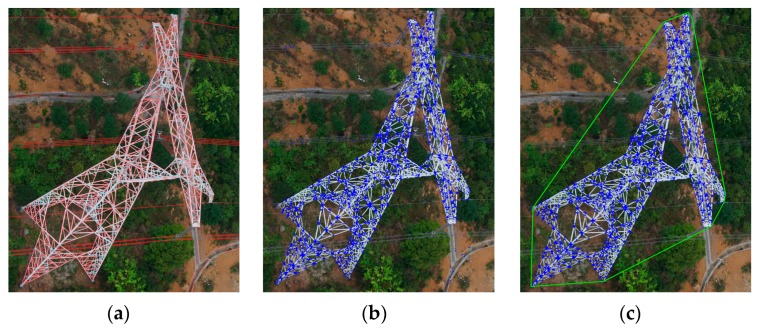
The results of intersection point computation and clustering (**a**–**c**).

The results of intersection point computation and clustering are shown in Figure 6. The rest of the line segments with red color after filtering with gradient symmetry are shown in Figure 6a. The blue points in Figure 6b are computed with the constraint mentioned above, and these points mainly cover the pylon. These points are clustered, and the final convex hull is shown in Figure 6c in green color. The region of the convex hull can be treated as the proposed region of the pylon.

## 5. 3D Reconstruction of Pylon Models

Once the pylons in the images have been detected, the pylon models are reconstructed automatically through a heuristic method. The reconstruction procedure consists of three steps: pylon type identification, pylon body reconstruction, and pylon head reconstruction. Before the pylon body reconstruction, the pylon type is identified using the IDSC method, and meanwhile, the correlation of 3D pylon legs and associated 2D line segments extracted in different images is obtained. After matching the line segments on the pylon body, the pylon body is reconstructed by fitting four principle legs and four side planes. As the structure of the pylon head is more complicated than the pylon body, it is difficult to match segments of pylon head to recover the 3D information. Unlike pylon body reconstruction, a MCMC method is employed to optimize the energy formulation to find the optimal parameters of pylon head through multi-view images.

### 5.1. Pylon Type Identification Based on Shape Matching

The pylon type can be identified by a shape matching method between the shape contours of projected models and shape contours of the pylon in the images. For complicated backgrounds and special structures of pylons, it is a difficult task to extract the rigid shape contours of the pylon precisely. As the intersection points in the detected region imply the distribution of pylon shape contours, it can be used to roughly extract the shape contours. 

**Figure 7:**
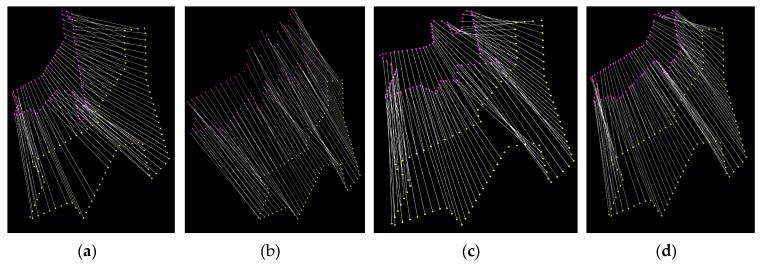
IDSC shape matching results. The red points are subsampled from the projected models with different types and the yellow points are subsamples from the same pylon in the image. (**a**) The same type of pylon is matched; (**b**–**d**) different types of pylon are matched.

After detecting the pylon using the DPM method, the proposed regions are confirmed and the intersection points of the cluster are used to extract pylon shape contours. First, the alpha shape method is applied to fit the shape contours from the intersection points. As the fitted shape contours contain short irregular segments which affect the shape matching result, the Douglas-Peucker method is adopted to simplify the shape contours. Second, the extrinsic and intrinsic parameters of cameras, the prior information of pylon 3D coordinates, and direction are used to project each model onto each visible image. The direction remains roughly consistent with the bisector of the angle which is computed from the lines of the two neighboring pylons connected to this pylon. Once the model has been projected onto the images, the connected external line segments are extracted as the shape contours of the projected model. The shape contours of projected model in the images are non-closed curves. Before projecting the model, the bottom endpoints of the four principle legs with the adjacent midpoints of bottom horizontal rectangle are connected to ensure that the shape contours of the projected model are closed (as shown in Figure 2a, the blue dotted lines). Third, the fixed number of points on the shape contours of projected model and pylon in image are subsampled. Finally, the IDSC method is adopted to identify the pylon type between the shape contours of projected model and pylon in image. The IDSC shape matching results are presented in Figure 7. For each type of model, the average shape matching cost is computed in each visible pylon images. The type of pylon T can be identified to be the same as the model for which the cost is the smallest. 

### 5.2. Pylon Body Reconstruction

The pylon body consists of four principle legs, two horizontal rectangular structures, and four side planes. Considering these features, the legs with a priori shapes implied by the pylon model are firstly matched. Then the remaining segments on the pylon body are matched with the constraint of coplanar condition. Finally, the parameters of the pylon body are analyzed through the least squares fitting algorithm.

#### 5.2.1. Pylon Body Matching Based on a Priori Shape and Coplanar Constraint

The IDSC method can identify the pylon type and meanwhile the matched sampling points on shape contours can be used to find the correlation of pylon 3D legs and extracted 2D line segments from different visible images. The pylon body line segment matching algorithm includes two steps. First, the four principle legs are matched using the correlation of pylon legs and the prior information regarding pylon body structures. Second, the parameters of the four side planes of the pylon body are fitted by the four matched legs and applied to match the rest of the line segments on the pylon body.

(1) Line segment matching for pylon legs

Let $I=\{I_{1},I_{2},\ldots ,I_{n}\}$ be the sequence of visible images of the pylon, each image contains the extracted 2D line segments $l^{I_{i}}=\{l_{1},l_{2},\ldots ,l_{n}\}$ of the pylon and the shape contours $s^{I_{i}}=\{s_{1},s_{2},\ldots ,s_{n}\}$. Let T be the identified type of pylon and $M_{t}$ be the corresponding pylon model. The pylon model consists of connected 3D line segments $L_{M_{t}}=\{L_{1},L_{2},\ldots ,L_{n}\}$. The shape contours of the projected pylon model $M_{t}$ on image $I_{i}$ are set to be $s_{M_{t}}^{I_{i}}=\{s_{1}^{\prime },s_{2}^{\prime },\ldots ,s_{n}^{\prime }\}$. The sampling points on the pylon shape contours of the image and the projected model are set to be $P^{I_{i}}=\{P_{1},P_{2},\ldots ,P_{k}\}$ and $P_{M_{t}}^{I_{i}}=\{P_{1}^{\prime },P_{2}^{\prime },\ldots ,P_{k}^{\prime }\}$, respectively.

For the identified type T of the pylon, the IDSC method matches the sampling points $P^{I_{i}}$ and $P_{M_{t}}^{I_{i}}$ between the two shape contours by minimizing the matching cost with the inner distance shape context descriptor. Once the minimized matching cost is determined, the matched sampling points’ correlation $f(P)=P^{I_{i}}⇄P_{M_{t}}^{I_{i}}$ can be obtained at the same time. The matched sampling points can be used to match the pylon legs. Given the two line segments $s_{k}$ and $s_{\bar{k}}^{\prime }$ of the pylon legs in $s^{I_{i}}$ and $s_{M_{t}}^{I_{i}}$, the matching score is defined as

(2) $$score(s_{k},s_{\bar{k}}^{\prime })=\max(\frac{N_{s_{k}\to s_{\bar{k}}^{\prime }}}{N_{s_{k}}},\frac{N_{s_{\bar{k}}^{\prime }\to s_{k}}}{N_{s_{\bar{k}}^{\prime }}}),$$

where $N_{s_{k}}$ stands for the total number of sampling points on line segment $s_{k}$ and $N_{s_{k}\to s_{\bar{k}}^{\prime }}$ means the number of sampling points on line segment $s_{k}$ that match the sampling points on line segment $s_{\bar{k}}^{\prime }$ using the IDSC method. If the score is above a fixed threshold value $T_{s}$ ($T_{s}=0.6$ in our experiments), the two line segments are accepted to be matched.

**Figure 8:**
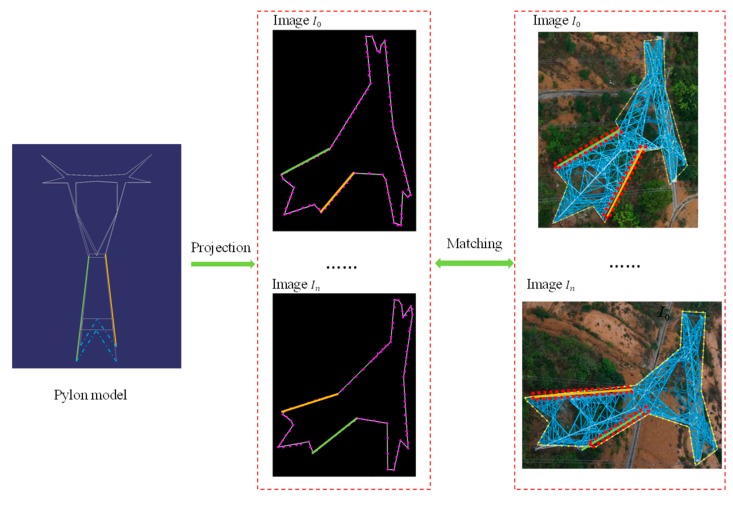
The workflow of correlation confirmation between 3D line segments of model and 2D line segments of pylon legs in images.

The line segments $s^{I_{i}}$ and $s_{M_{t}}^{I_{i}}$ are the intermediate objects that are used to find the correlation between the 2D line segments in $l^{I_{i}}$ and the 3D line segments of the pylon legs in $L_{M_{T}}$ (as shown in Figure 8). For each matched line segment $s_{k}$ in $s^{I_{i}}$, a buffer area $R_{1}$, which is same as the R mentioned in Section 4.2, and the parameter $T_{w}$ in this situation is set to be 50, is generated to find the associated line segments in $l^{I_{i}}$. If the line segment $l_{n}$ in $l^{I_{i}}$ is in this buffer area and the angle between $l_{n}$ and $s_{k}$ is less than a threshold value $T_{a}$($T_{a}=20{}^{\circ}$ in our experiments), $l_{n}$ is supposed to be affiliated with $s_{k}$. With the matched line segments between $s^{I}$ and $s^{M}$, the 2D line segments of $l^{I_{i}}$ from different images that belong to the same 3D line segment of the pylon legs in $L_{M_{T}}$ can be identified. As the projection matrix $P^{i}$ is known, the line segments from different images associated with a same 3D line segment of pylon legs in $L_{M_{T}}$ can be triangulated to compute the 3D line segment coordinates of pylon legs using an epipolar-guided line matching algorithm similar to that used in [14,30]. Let $l_{k}^{I_{i}}$ and $l_{\bar{k}}^{I_{j}}$ be the line segments of image $I_{i}$ and image $I_{j}$, and they both belong to the same 3D line segment in $L_{M_{T}}$. The intersection points $x_{1}$ and $x_{2}$ are computed between the epipolar lines of the endpoints of $l_{k}^{I_{i}}$ and the infinite lines passing through the line segment $l_{\bar{k}}^{I_{j}}$, and the intersection points $x_{1}$ and $x_{2}$ are collinear with the endpoints $p_{\bar{k}}^{I_{j}}$ and $q_{\bar{k}}^{I_{j}}$ of the line segment $l_{\bar{k}}^{I_{j}}$. If the matching score between line segment $l_{k}^{I_{i}}$ and $l_{\bar{k}}^{I_{j}}$ is above a fixed threshold τ (τ=0.25 in all our experiments), the intersection points and the endpoints of $l_{k}^{I_{i}}$ can be triangulated to generate the 3D line segment coordinates. The matching score is defined as

(3) $$s(l_{k}^{I_{i}},l_{\bar{k}}^{I_{j}})=\frac{\mathrm{inner}(p_{\bar{k}}^{I_{j}},q_{\bar{k}}^{I_{j}},x_{1},x_{2})}{\mathrm{outer}(p_{\bar{k}}^{I_{j}},q_{\bar{k}}^{I_{j}},x_{1},x_{2})}$$

where the inner($p_{\bar{k}}^{I_{j}},q_{\bar{k}}^{I_{j}},x_{1},x_{2}$) and outer($p_{\bar{k}}^{I_{j}},q_{\bar{k}}^{I_{j}},x_{1},x_{2}$) are the Euclidean distance between the inner and the outer pair of the four collinear points ($p_{\bar{k}}^{I_{j}}$, $q_{\bar{k}}^{I_{j}}$, $x_{1}$ and $x_{2}$), respectively. As there are many fractured 2D line segments that belong to the same 3D line segment of the pylon legs, the RANSAC (Random Sample Consensus) [31] method is adopted to remove outliers and the final 3D line segment coordinates are computed by least squares fitting technique.

**Figure 9:**
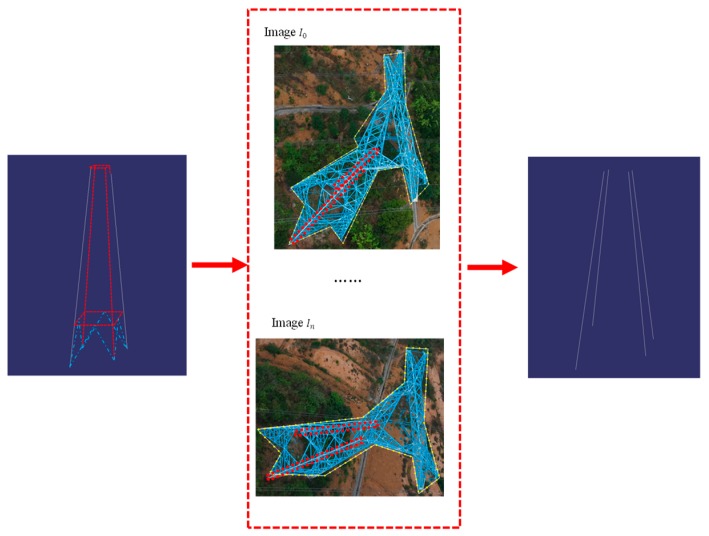
The workflow of the rest of the pylon leg matching.

Through the above method, the pylon legs which are located inside the shape are still unmatched. As shown in Figure 2a, there are two horizontal rectangle structures on pylon body and the rectangle structures are intersected with the four pylon legs. For the unmatched pylon legs, their hypothetical coordinates can be initially generated with constraint of horizontal rectangle structures and the matched 3D line segments of pylon legs. Then these hypothetical 3D line segments are projected onto each visible image. For the projected hypothetical 3D line segment of the pylon legs, the buffer area $R_{1}$ is generated again to find the corresponding 2D line segments in $l^{I_{i}}$ as mentioned above. Finally, with the epipolar-guided line matching algorithm and RANSAC method, the accurate 3D coordinates of unmatched pylon legs are computed. Figure 9 illustrates the workflow of the rest of the pylon leg matching algorithm.

(2) Line segment matching for the pylon body

After matching the four pylon legs, the four side plane parameters can be fitted using the matched pylon leg coordinates by least squares fitting technique. As the line segments of pylon body are distributed on the four side planes, the four side planes are used to match the rest line segments of the pylon body. First, the matched pylon legs are projected in each visible image and the convex hull of the projected pylon legs are computed. The 2D line segments in the convex hull of each visible image are used to generate 3D line segments of pylon body. Let $f=\{f_{1},f_{2},f_{3},f_{4}\}$ be the four side planes. Given a 2D line segment $l_{k}\in l^{I_{i}}$ where $l_{k}$ is in the convex hull, the costs of $l_{k}$ to be labeled with $f_{i}(i=1,2,3,4)$ are respectively computed. If $l_{k}$ is labeled with the plane $f_{i}$, the two rays passing through the endpoints of $l_{k}$ are used to intersect with $f_{i}$ and the 3D coordinates of $l_{k}$ are computed. Then the 3D line segment of $l_{k}$ is projected onto each image $I_{j}\in I-I_{i}$ and it is found whether there exists a line segment in the buffer area R of the projected line segment $l_{p}^{I_{j}}$. Then the cost of $l_{k}$ to be labeled with $f_{i}$ is computed using the function:

(4) $$cost(l_{k},f_{i})=\frac{\sum_{j=0}^{N_{v}}\log(\frac{\exp(-\max(dist_{j}))}{\sqrt{2\pi }})+\exp(-angle_{j})}{N_{v}}(N_{v}>0)$$

where $N_{v}$ is the number of images in which the line segment $l_{k^{\prime }}^{\prime }$ is found in the buffer area R of $l_{p}^{I_{j}}$; the $\max(dist_{j})$ is the max distance between the endpoints of $l_{k^{\prime }}^{\prime }$ and $l_{p}$; the $angle_{j}$ is the angle between the two segments. The $l_{k}$ is assumed to be coplanar with the plane $f_{i}$ if the cost labeled with $f_{i}$ is the minimum. After finding the plane $f_{i}$ that is coplanar with $l_{k}$, the 2D line segments in different images that are in the buffer area R of $l_{p}^{I_{j}}$ are triangulated to compute the 3D coordinates with the epipolar-guided line matching algorithm, and the RANSAC method is applied to remove outliers.

#### 5.2.2. Pylon Body Parameter Estimation

The pylon body has the features of symmetry and perpendicularity. The parameters of the four side planes fitted above only using the four pylon legs coordinates need to be adjusted in consideration of these features. After matching 3D line segments of pylon body, we follow the approach of Li and Chen et al., who proposed an adjustment model using least squares fitting with additional parameters to fit the four side plane parameters. Linearization and iterative solutions are adopted to solve the model. The four side plane parameters fitted in the previous section are set to be initial values. The four pylon legs are then computed by intersecting the four side planes.

There are a number of horizontal line segments distributed in the pylon body. To compute the height and position of the pylon body, the two horizontal rectangles defined in the model should be found in the horizontal line segments. First, the maximum height $H_{\max}$ and minimum height $H_{\min}$ of these horizontal line segments are computed. Second, the histogram of the height $H=H_{\max}-H_{\min}$ with a certain bin width ΔH is generated. In addition, the horizontal line segments whose height can be located are grouped in the same histogram bin. The average height of the group with the maximum value is regarded as the top horizontal rectangle height. Third, the total length of the group whose average height is in the range $\left(H_{\min},H_{\min}+\frac{1}{3}H\right)$ is computed. As most of the line segments in a group overlap with other line segments, for such line segments, a merging strategy needs to be conducted before the total length is computed. The average height of the group is used to intersect with the four pylon legs and the two neighboring intersection points are set to be the new line direction of the line segments which are on the same side plane. For the overlapping line segments, all the endpoints of these line segments are projected into the new line and the overlapping line segments are replaced with the line segment defined by the two outmost points. After merging the overlapping line segments, the total length of the left line segments in the group is calculated. The average height of the group with maximum length value is regarded as the bottom horizontal rectangle height. Finally, the heights of the top and bottom horizontal rectangles are used to intersect with the four pylon legs to compute the parameters of the two rectangles. The horizontal position of the pylon body is derived from the average center of the two horizontal rectangles. In addition, the height of the position of the pylon body is set to be the same as the height of the bottom horizontal rectangle. The direction of the line defined by the two midpoints of the bottom horizontal rectangle edges that are at the front and back sides is treated as the orientation of the pylon. The detailed algorithm is shown below (Algorithm 1):
**Algorithm 1.** Pylon Body Parameter Estimation**Input:**  matched horizontal 3D line segments of pylon body; fitted four pylon legs **Output:**  parameters of pylon body 1:Compute the maximum height $H_{\max}$ and minimum height $H_{\min}$ of the line segments2:Generate the histogram of the height $H=H_{\max}-H_{\min}$ with a certain bin width ΔH3:Group the line segments of which height locate in the same histogram bin4:Find the maximum height of the group and regard its average height as the top horizontal rectangle height5:Compute the total length of the groups of which height is in the range $\left(H_{\min},H_{\min}+\frac{1}{3}H\right)$6:Fine the maximum length of the group and regard its average height as the bottom horizontal rectangle height7:Intersect the four pylon legs with the top and bottom horizontal rectangle height8:Compute the height, position and direction of pylon body

### 5.3. MCMC-Based Pylon Head Reconstruction

The structures of pylon heads are more complicated than those of pylon bodies. To match the 3D line segments of pylon head from multi-view images is a great challenge. Instead of recovering the pylon head parameters from the matched 3D line segments like the pylon body, the reconstruction of the pylon head is transformed to be an optimization procedure, sampling from images using the Markov Chain Monte Carlo (MCMC) method. Firstly, the parameters of the pylon body, position, and orientation of the pylon which have been confirmed are used to update the pylon model coordinates. Then, the pylon model is projected onto all the visible images to measure the similarity between the line segments of projected pylon head and the extracted line segments of the pylon in the images. The MAP solution is approximated to optimize the parameters of the pylon head using the MCMC method.

The parameters of the pylon head are estimated using the MCMC procedure, which is based on the Metropolis-Hastings (MH) algorithm. The continuous parameter space of the pylon head is denoted as θ and the target distribution of the projected pylon head model M in the line segments $l^{I}$ of multi-images is modeled as an exponential distribution:

(5) $$P(\theta |l^{I})\sim e^{-J(\theta )}$$

where J(θ) is the similarity measure between the projected pylon head model $l^{M}$ and $l^{I}$. The maximum posterior probability solution is adopted to approximate θ from the distribution $P(\theta |l^{I})$, using the MH algorithm. For a given parameter state $\theta^{†}$ of the pylon head, the pylon head 3D coordinates of line segments in the model are updated and the line segments are projected onto each of the visible images. For each projected line segment $l_{k}^{M}$ of the pylon head, the line segments $N_{l}$ ($N_{l}\in l^{I_{i}}$) which are in the buffer area R of $l_{k}^{M}$ are found. The $N_{l}$ are fitted as a single line segment $l_{f}$ by the least squares method. Then $l_{k}^{M}$ and $l_{f}$ are used to compute the posterior probability. $J(\theta^{†})$ is determined as:

(6) $$J(\theta^{†})=\sum_{k=0}^{A}\frac{1}{B}\{\sum_{i=0}^{B}\left(\log(\exp(-angle_{k,i})+1.2*\exp(-dist_{k,i}\right)+0.6*\exp(overlap_{k,i}-1))\}$$

where A is the number of line segments in the pylon head model and B is the number of visible images; the $angle_{k,i}$ is the angle between $l_{f}$ and $l_{k}^{M}$ in image $I_{i}$; the $dist_{k,i}$ is the max distance between $l_{f}$ and $l_{k}^{M}$ in image $I_{i}$; the $overlap_{k,i}$ means the overlap ratio between $l_{f}$ and $l_{k}^{M}$ in image $I_{i}$. The overlap ratio is computed as follows:

(1) Select the longest line segment between $l_{f}$ and $l_{k}^{M}$ as the new target line $l_{t}$;
(2) Project the endpoints of the other line segment onto $l_{t}$;
(3) Compute the distance of inner two endpoints $d_{i}$ and the distance of the two outward endpoints $d_{o}$, the ratio $\frac{d_{i}}{d_{o}}$ is treated as the overlap ratio.

In our case, the parameters of pylon body remain fixed in each iteration step and the parameters of the pylon head are chosen randomly from their possible ranges. The MCMC algorithm proceeds by changing the current parameter state θ to a new state $\theta^{†}$ with an acceptance probability

(7) $$A(\theta \to \theta^{†})=\min(1,\frac{P(\theta^{†}|L)u(\theta^{†}\to \theta )}{P(\theta |L)u(\theta \to \theta^{†})})$$

where the $u(\theta^{†}\to \theta )$ is the proposed distribution of the parameters, which is a uniform distribution in a range defined by the user.

## 6. Experiments and Results

### 6.1. Datasets

The proposed method is applied to reconstruct the pylons from oblique UAV images acquired in Shenzhen, China. A multi-rotor UAV is used to fly above the high transmission line on both sides of the pylon, and the flight trajectory is a closed loop. The same oblique photogrammetric system and flight configuration of UAV found in reference [3], with only a front digital camera (camera mode: Sony RX1R, focal length: 35mm, complementary metal oxide semiconductor (CMOS) sensor: 24 Mpixel (6000 × 4000 pixels), physical camera dimensions: 35.8 mm × 23.9 mm), are applied for the photography of the high transmission line corridor. The flight heights listed in Table 1 are relative to the position from which the UAV takes off. 

**Figure 10:**
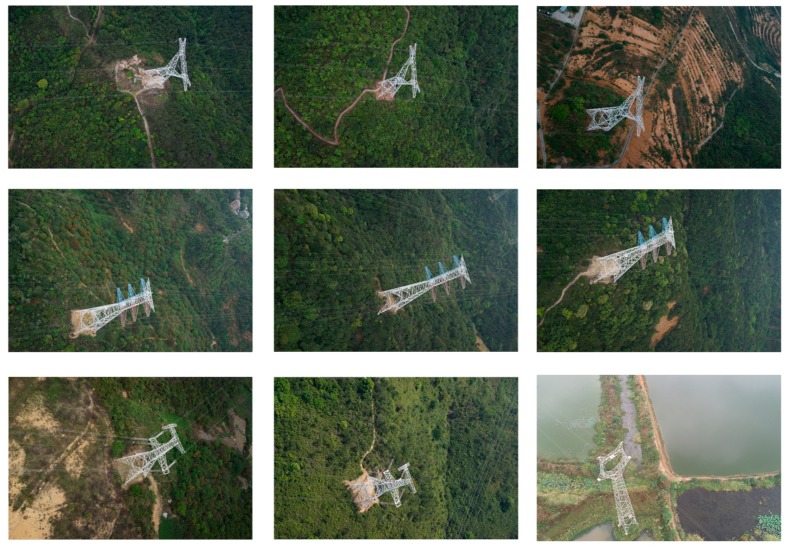
The images of pylon.

Five flight datasets which contain 15 pylons are applied in the experiments. There are nine images from the 15 pylons that are presented in Figure 10. The first four flight datasets are in mountain areas and the last one is near waters. The camera calibration and image orientation were performed using the free software MicMac [32]. The experiments were all conducted on Intel Core i7 CPU machine with NVidia GTX960 graphic card, 16GB RAM and 64-bit Windows 7 OS and implemented by C++.

### 6.2. The Performance Analysis of Pylon Detection

**Figure 11:**
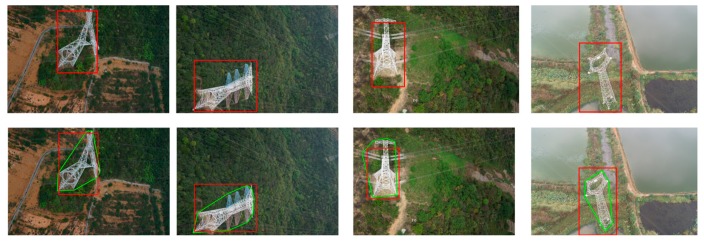
The results of pylon detection. The first row is the result of pylon detection by the DPM method and the second is the proposed method. The red rectangle is the detected bounding box location of the pylon. In the second row, the green polygon is the proposed region.

In this experiment, 120 images are selected to manually label the location of pylons as training data. In addition, five flight datasets from UAV images of different transmission lines comprise the test data to examine the efficiency of pylon detection. According to the resolution of images and the structure of the pylons, the parameters of $T_{d}$, $T_{w}$ and $T_{Num}$ are set to be 20, 60 and 1000, respectively; the two parameters of DBSCAN (*Eps* and *MinPts*) are set to be 50 and 20; and the cluster density threshold is set to be 0.01. The four threads are used in the procedure of pylon detection with OpenMP technology. The four images are randomly selected as representative, are shown in Figure 11. 

The pylon detection results with the proposed method and the DPM method are listed in Table 2. N is the number of images, the correctness and completeness are evaluated with the same equation as in [10].

(8) $$\{\begin{array}{c} Completeness=TP/(TP+FN)\\ Correctness=TP/(TP+FP) \end{array}$$

where TP is the number of correctly detected pylons, with the loU (Intersection over Union) score being bigger than 0.5; FP is the number of wrongly detected pylons (the loU score is less than 0.5); and FN is the number of undetected pylons. For the purpose of pylon reconstruction, only the images covering the whole pylon are considered for evaluating correctness and completeness. The results indicate that the cost in time and maximum consumed memory for pylon detection suing the proposed are significantly improved. The cost of time and maximum consumed memory of the proposed method varies based on differences in the surroundings of the pylon and the resolution of images, while the DPM method is stable, as it uses a sliding window to detect the objects. The experiment shows that the efficiency of pylon detection can be improved by computing the proposed regions and directly detecting the pylon in the proposed regions instead of using the sliding window method. The correctness of the proposed method is much higher than the DPM method, because in the process of computing the proposed regions, the wrongly detected regions are filtered out. The proposed method can retains the same completeness as the DPM method.

### 6.3. The Recognition of Pylon Type

In this experiment, fifteen pylons selected from five sets of flight data are tested for the recognition of pylon type. All the pylon types can be correctly identified by the IDSC method. The eight randomly selected matching costs between the extracted pylon shape contours and the projected pylon models are listed in Table 3.

The results of the experiment suggest that the matching cost of the same pylon type is much lower than that of different pylon types in the model, which can robustly identify the right model type. Although the shape contours of the pylon in images extracted by the alpha shape method are not rigidly fitted to the real shape of pylon, the IDSC algorithm can still distinguish the different pylon types robustly.

### 6.4. 3D Model Reconstruction of Pylon Body and Head

**Figure 12:**
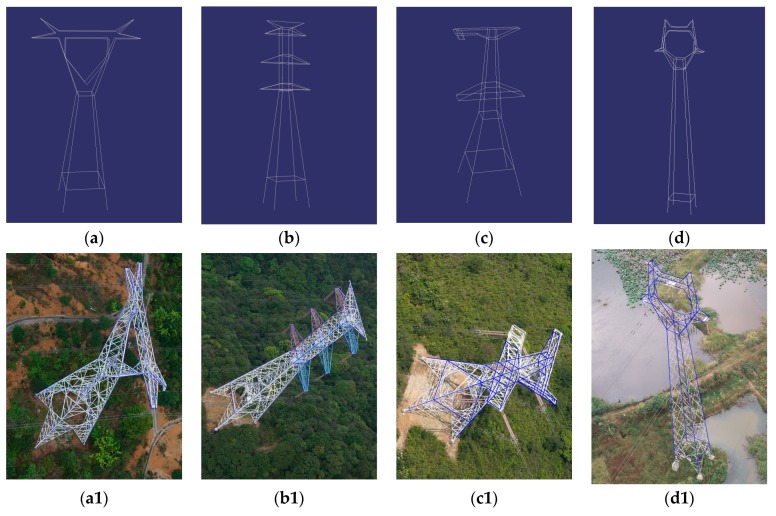

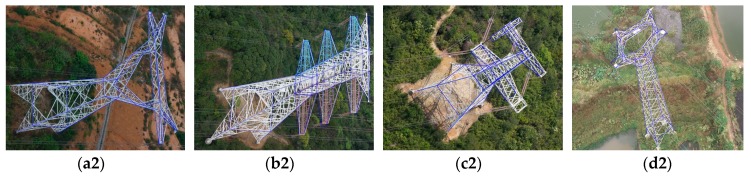
Samples of four type pylon reconstruction results. (**a**–**d**) are the reconstructed pylon models of Type 1, Type 2, Type 3 and Type 4, respectively. The second and third rows are the projected model from different image perspectives.

In this paper, we automatically reconstructed fifteen pylons of four typical types using the proposed method. To visually check the reconstructed model, the line segments of the pylon model are projected onto each visible image, as shown in Figure 12.

To evaluate the pylon body fitting accuracy, the adjacent image pairs of pylons are used to measure the endpoints of the mainframe line segments on the pylon body using our self-developed stereo-measurement software. Then the average distances from manually measured line segment coordinates to the corresponding side plane are calculated. To evaluate the accuracy of horizontal position and orientation accuracy, some horizontal rectangular structures of the pylon body are manually measured to compute the horizontal position and orientation. $\delta_{x}$ and $\delta_{y}$ stand for the difference value of x position and y position between the automatically extracted horizontal center position and the manually measured horizontal center position, respectively; $\delta_{d}$ represents the difference value of the pylon direction. These are defined by the equation:

(9) $$\{\begin{array}{c} \delta_{x}=|m_{x}-a_{x}|\\ \delta_{y}=|m_{y}-a_{y}|\\ \delta_{d}=|m_{d}-a_{d}| \end{array}$$

where $m_{x}$ and $m_{y}$ stand for the manually measured horizontal center position of x and y; $m_{d}$ is the manually measured direction of the pylon; $a_{x}$ and $a_{y}$ stand for the automatically extracted horizontal center position of x and y; $a_{d}$ is the automatically computed direction of the pylon. The SD stands for standard deviation value.

Table 4 gives the result of fifteen pylons with four types. The results indicate that the average distance of line segments to the four side planes is about 0.188 m. The precision of pylon horizontal position reached 0.07 m, and that of pylon orientation reached 0.65°. 

In this experiment, fifteen pylons of the four types mentioned above are selected to test the accuracy of pylon head reconstruction. The 3D coordinates of the mainframe line segments on the pylon head are manually measured with our self-developed stereo-measurement software. To quantitatively evaluate the accuracy of the pylon head reconstruction results, the average distance $D_{ave}$ and RMSE (root-mean-square error) of manually measured line segments to the reconstructed model are calculated according to Equations (10) and (11) respectively:

(10) $$D_{ave}=\frac{1}{2n}\sum_{i=1}^{n}\sum_{j=1}^{2}\left(dist(m_{i,j},L)\right),$$

(11) $$\delta_{rmse}=\sqrt{\frac{1}{2n}{\sum_{i=1}^{n}\sum_{j=1}^{2}\left(dist(m_{i,j},L)\right)}^{2}}$$

where n is the number of manually measured line segment; $m_{i,j}$ is the jth (j=1,2) endpoint of the ith (0<i≤n) manually measured line segment; $dist(m_{i,j},L)$ is the closest distance of $m_{i,j}$ to the model L.

In practice, the MCMC algorithm is run for 10,000 iterations and the sample with the highest probability is regarded as the approximate estimation of the MAP solution. The number of unknown parameters in type 1, type 2, type 3 and type 4 are 16, 20, 13 and 17, respectively. The accuracy of head reconstruction is listed in Table 5. The average distance $D_{ave}$ of the pylon head is 0.316 m on average, while the RMSE is 0.372 m.

## 7. Discussion

This research introduces an automatic model-driven method for pylon reconstruction from oblique UAV images. The influence of α value for shape contours extraction and the influence factors of pylon reconstruction are discussed in this section.

### 7.1. The Influence of α Value in Alpha Shape for Contour Extraction

The shape contours of pylon extraction is a key step for pylon reconstruction. It is closely related to pylon type identification, and the pylon reconstruction process. If there is great deformation between the extracted shape contours and the real contours of pylon, the pylon type identification and reconstruction process may fail. The parameter α in alpha shape controls the desired level of detail. For each real number α, when α>0, it is a closed disk of radius 1/α. To determine the impact of the parameter α in alpha shape method on the shape contour extraction results, four α values are applied in images with different pylon types, and the results are shown in Figure 11.

**Figure 13:**
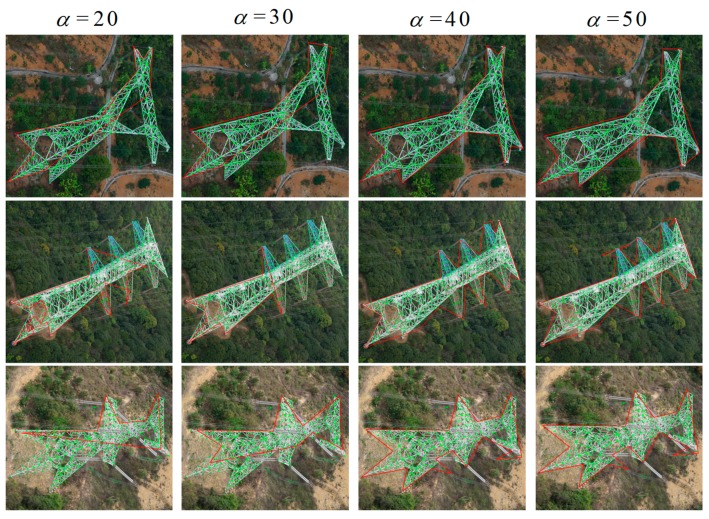
The results of alpha shape method with different α values. The red line segments in images are the outer contours extracted by alpha shape method. The green points are the clustered intersection points of extracted line segments in images.

From Figure 13, it can be seen that, when the α value is less than 40, the extracted shape contours only contain partial region of the pylon, which cannot be used to identify the pylon type and reconstruct the pylon model. However, if the α value is approaching infinite, the extracted shape contours become convex hull, which cannot represent the detail features of pylon shape. Thus, the α value should be cautiously selected. In our experiments, it can be found that the α value in the range (40, 60) can obtain a better result.

### 7.2. The Influence Factors of Reconstruction Errors

Although some details of the pylon shape may be missing and there are some deformed parts in the extracted shape contours, the IDSC method can still identify the right pylon type and match the pylon legs successfully. Except for the α value in the pylon shape contour extraction step, which can lead to failure of pylon reconstruction, there are still other factors that influence the pylon reconstruction errors.

**Figure 14:**
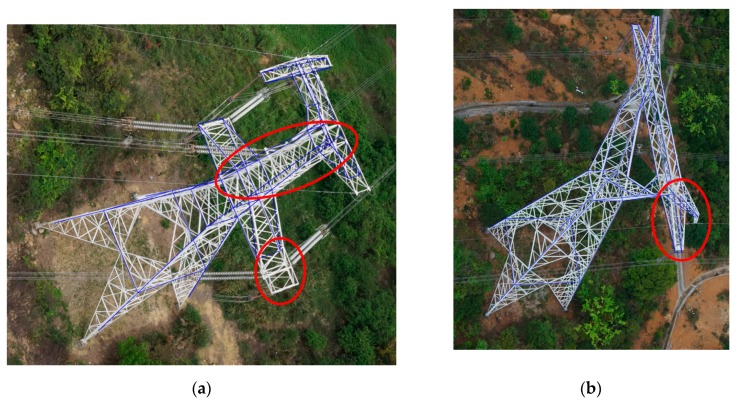
Examples of reconstruction errors (**a**,**b**).

(1) The pylon consists of metal structures with a certain width. The edges of such structures in different images are not in strictly the same position in the object coordinate space. In this respect, the width of metal structures increase the errors between the reconstructed pylon model fitting by the edges and the manually measured line segments by stereo-measurement software.
(2) The pylon is usually vertical. However, due to some complicated reasons, the pylon may be inclined. The model-driven method cannot adapt this situation. In addition, if there are some small irregular parts that are not defined in the pylon library, the proposed method cannot reconstruct the irregular parts. As shown in Figure 14a, the pylon head is inclined, which causes the related cross arms in the pylon head to deviate from the projected line segments of the reconstructed pylon model. In addition, the right bottom cross arm is unsymmetrical with the left one, there is an affiliated structure connected with the cross arm. The affiliated structure in different pylons with the same type always changes, which is improper for defining such structures in the model. One solution to this problem is to introduce a primitive-based model library to enhance the adaptability of the pylon model.
(3) For the small parts in the pylon head, the 2D line segments are visible only in a few images. These small parts contribute little to the energy term calculation, which affects the final accuracy. In addition, the MCMC sampler usually finds an approximate global optimization, but not an absolute optimization in the finite iteration. In addition, the uncertainty of the line segments’ endpoints and the occlusion issues also affect the fitting accuracy of pylon head. As shown in Figure 14b, in the pylon head of type 1, the small structures are obscured by the others, affecting the reconstructed accuracy.
(4) The images of the fifteen pylons in the experiments are collected in well-conditioned situations. However, for some adverse situations (such as encroaching vegetation, weather and light conditions, the pylon in images overlapped in the direction of the line of sight), the problems of serious occlusion and bad image quality would affect the pylon reconstruction results and could even lead to the failure of pylon reconstruction.

## 8. Conclusions

In this paper, a new model-driven framework for pylon reconstruction from oblique UAV images is proposed. This framework mainly consists of three parts: efficient pylon detection, pylon body reconstruction, and pylon head reconstruction. Considering the features of pylons in UAV images, the proposed regions which may contain pylons are obtained. The DPM method is applied to detect the pylon only in these proposed regions, instead of the whole image region, which improves the efficiency of pylon detection and saves memory. Once the shape contours of the pylons in the images are extracted, the IDSC method is employed to identify the pylon type, and meanwhile, for the pylon legs, the correlation of the extracted 2D line segments in the different images and the 3D line segments of the pylon model is confirmed in order to recover the 3D line coordinates. With the constraint of the pylon body structures, the line segments on the pylon body are matched. Then the pylon body is reconstructed by fitting the four principle legs and four side planes. As shown in the experiments, the average distance of line segments to the four side planes of pylon body is about 0.188 m. The precision of pylon horizontal position reached 0.07 m and the pylon orientation reached 0.65°. For the pylon head reconstruction, a MCMC sampler is designed to approximate the parameters of pylon head. In each sampling step, the parameters of the pylon body are kept fixed, which reduces the parameters for optimization. The pylon head reconstruction experiments demonstrate that the average distance of the measured line segments from the reconstructed pylon head model is 0.316 m. Experiments on the five flight datasets suggest that the proposed method can reconstruct the pylon body and pylon head automatically.

However, the proposed method also has several limitations. First, the shape contours of pylon extracted by the alpha shape method cannot strictly fit the edges of pylon shape. In recent years, deep learning methods have achieved promising results in object detection and segmentation fields. The deep learning method would be applied for the pylon detection and segmentation in our future work. Second, as the five flight data in our experiments contains four types of pylon, the model library only defines four types of pylon, which is able to satisfy the pylon reconstruction of the five flight data. To improve the adaptive ability, more types of pylon should be added to the library. Third, another limitation is that the proposed model-driven method is only applicable for pylons that fit the parametric model, which is also one limitation of most model-driven methods. As the pylon types vary greatly in different situations, a more generalized parametric pylon library, such as a primitive-combining model, could be considered. Finally, our method only reconstructs the main line structure without considering the material width and auxiliary structures. In future work, the ALS data and UAV images will be combined together to reconstruct higher accuracy models in more detail.

## Figures and Tables

**Table 1:** Detailed information for flight configuration.

Dataset	1	2	3	4	5
**Flying Height (m)**	137	160	115	103	70
**Number of Pylon**	4	2	4	2	3
**Voltage (Kv)**	500	500	500	500	220

**Table 2:** Pylon detection results.

Dataset	N	Time (h)		Max Memory (Gb)		Correctness		Completeness	
		DPM	Ours	DPM	Ours	DPM	Ours	DPM	Ours
1	138	2.87	0.13	13.13	1.14	78%	100%	88.64%	88.64%
2	138	2.77	0.15	13.13	1.45	95.65%	100%	97.78%	97.78%
3	257	5.33	0.22	13.13	1.67	76.19%	91.43%	94.12%	94.12%
4	100	2.08	0.14	13.13	1.56	82.5%	100%	94.82%	94.82%
5	119	2.52	0.11	13.13	1.18	73.33%	100%	93.94%	93.94%

**Table 3:** Different shape matching costs.

Pylon No.	Model 1	Model 2	Model 3	Model 4	Type
1	26.212	61.514	54.564	34.930	1
2	29.560	53.282	66.793	43.125	1
3	63.598	31.569	48.270	46.996	2
4	61.625	35.785	39.992	43.778	2
5	64.969	49.538	35.713	43.641	3
6	66.170	69.879	47.158	51.902	3
7	59.299	57.046	44.680	29.057	4
8	47.973	46.351	47.314	39.647	4

**Table 4:** The accuracy of pylon body reconstruction.

Pylon No.	Pylon Type	Residuals	$\delta_{x}$ (m)	$\delta_{y}$ (m)	$\delta_{d}$ (°)
1	1	0.164	0.126	0.030	0.781
2	1	0.074	0.001	0.028	0.778
3	1	0.097	0.036	0.046	0.783
4	3	0.240	0.025	0.026	0.794
5	2	0.316	0.005	0.005	0.087
6	2	0.313	0.007	0.026	0.088
7	1	0.246	0.121	0.113	0.789
8	1	0.137	0.049	0.032	0.781
9	1	0.134	0.074	0.012	0.769
10	3	0.268	0.124	0.139	0.081
11	2	0.161	0.073	0.068	0.724
12	2	0.186	0.114	0.120	0.789
13	4	0.230	0.215	0.004	0.732
14	4	0.072	0.001	0.008	0.879
15	4	0.175	0.031	0.117	0.840
Mean	--	0.188	0.067	0.052	0.646
SD	--	0.079	0.062	0.047	0.292

**Table 5:** The accuracy of pylon head reconstruction.

Pylon No.	$D_{ave}$ (m)	RMSE (m)
1	0.387	0.424
2	0.275	0.321
3	0.336	0.352
4	0.305	0.345
5	0.282	0.343
6	0.381	0.406
7	0.408	0.426
8	0.275	0.309
9	0.296	0.345
10	0.302	0.340
11	0.352	0.407
12	0.277	0.322
13	0.308	0.425
14	0.261	0.391
15	0.293	0.425
Mean	0.316	0.372
SD	0.046	0.044

## References

[1] Zhang Y., Yuan X., Li W., Chen S. (2017). Automatic power line inspection using uav images. Remote Sens..

[2] Airborne and Terrestrial Laser Scanning. https://research.utwente.nl/en/publications/airborne-and-terrestrial-laser-scanning-2.

[3] Jiang S., Jiang W., Huang W., Yang L. (2017). Uav-based oblique photogrammetry for outdoor data acquisition and offsite visual inspection of transmission line. Remote Sens..

[4] Musialski P., Wonka P., Aliaga D.G., Wimmer M., Van Gool L., Purgathofer W. (2013). A survey of urban reconstruction. Comput. Graph. Forum.

[5] Jiang S., Jiang W. (2018). Efficient sfm for oblique uav images: From match pair selection to geometrical verification. Remote Sens..

[6] Jiang S., Jiang W. (2019). Efficient match pair selection for oblique uav images based on adaptive vocabulary tree. ISPRS J. Photogramm. Remote Sens..

[7] Von Gioi R.G., Jakubowicz J., Morel J.-M., Randall G. (2012). LSd: A line segment detector. Image Process. On Line.

[8] Akinlar C., Topal C. (2011). EDlines: A real-time line segment detector with a false detection control. Pattern Recognit. Lett..

[9] Ling H., Jacobs D.W. (2007). Shape classification using the inner-distance. IEEE Trans. Pattern Anal. Mach. Intell..

[10] Li Q., Chen Z., Hu Q. (2015). A model-driven approach for 3d modeling of pylon from airborne lidar data. Remote Sens..

[11] Guo B., Huang X., Li Q., Zhang F., Zhu J., Wang C. (2016). A stochastic geometry method for pylon reconstruction from airborne lidar data. Remote Sens..

[12] Chen S., Wang C., Dai H., Zhang H., Pan F., Xiaohuan X., Yan Y., Wang P., Yang X., Zhu X. (2019). Power pylon reconstruction based on abstract template structures using airborne lidar data. Remote Sens..

[13] Zhou R., Jiang W., Wei H., Bo X., Jiang S. (2017). A heuristic method for power pylon reconstruction from airborne lidar data. Remote Sens..

[14] Hofer M., Maurer M., Bischof H. (2016). Efficient 3d scene abstraction using line segments. Comput. Vis. Image Underst..

[15] Fryskowska A. (2019). Improvement of 3d power line extraction from multiple low-cost uav imagery using wavelet analysis. Sensors.

[16] Sampedro C., Martinez C., Chauhan A., Campoy P. (2014). A supervised approach to electric tower detection and classification for power line inspection. Proceedings of the 2014 International Joint Conference on Neural Networks (IJCNN).

[17] Jalil B., Leone G., Martinelli M., Moroni D., Pascali M., Berton A. (2019). Fault detection in power equipment via an unmanned aerial system using multi modal data. Sensors.

[18] Han J., Yang Z., Zhang Q., Chen C., Li H., Lai S., Hu G., Xu C., Xu H., Wang D. (2019). A method of insulator faults detection in aerial images for high-voltage transmission lines inspection. Appl. Sci..

[19] Hoiem D., Efros A.A., Hebert M. (2005). Automatic photo pop-up. ACM Trans. Graph..

[20] Košecká J., Zhang W. (2005). Extraction, matching, and pose recovery based on dominant rectangular structures. Comput. Vis. Image Underst..

[21] Barinova O., Konushin V., Yakubenko A., Lee K., Lim H., Konushin A., Forsyth D., Torr P., Zisserman A. (2008). Fast automatic single-view 3-d reconstruction of urban scenes. Proceedings of the Computer Vision—ECCV 2008: 10th European Conference on Computer Vision.

[22] Saxena A., Sun M., Ng A.Y. (2009). Make3d: Learning 3d scene structure from a single still image. IEEE Trans. Pattern Anal. Mach. Intell..

[23] Werner T., Zisserman A., Heyden A., Sparr G., Nielsen M., Johansen P. (2002). New techniques for automated architectural reconstruction from photographs. Proceedings of the Computer Vision—ECCV 2002: 7th European Conference on Computer Vision.

[24] Dick A.R., Torr P.H.S., Cipolla R. (2004). Modelling and Interpretation of Architecture from Several Images. Int. J. Comput. Vis..

[25] Xiao J., Fang T., Zhao P., Lhuillier M., Quan L. (2002). Image-based street-side city modeling. Proceedings of the 10th European Conference on Computer Vision.

[26] Felzenszwalb P.F., Girshick R.B., McAllester D., Ramanan D. (2010). Object detection with discriminatively trained part-based models. IEEE Trans. Pattern Anal. Mach. Intell..

[27] Katahara S., Aoki M. (2002). Face parts extraction windows based on bilateral symmetry of gradient direction. Proceedings of the 8th International Conference, CAIP’99.

[28] Li K., Yao J. (2017). Line segment matching and reconstruction via exploiting coplanar cues. ISPRS J. Photogramm. Remote Sens..

[29] Ester M., Kriegel H.-P., Sander J., Xu X. (1996). A density-based algorithm for discovering clusters in large spatial databases with noise. KDD.

[30] Hofer M., Maurer M., Bischof H. (2014). Improving sparse 3d models for man-made environments using line-based 3d reconstruction. Proceedings of the International Conference on 3d Vision.

[31] Fischler M.A., Bolles R.C. (1987). Random sample consensus: A paradigm for model fitting with applications to image analysis and automated cartography. Read. Comput. Vis..

[32] An Open Source Bundle Adjusment Software for Automatic Calibration and Orientation of Set of Images. https://www.semanticscholar.org/paper/APERO%2C-AN-OPEN-SOURCE-BUNDLE-ADJUSMENT-SOFTWARE-FOR-Deseilligny-Clery/bd88800990aa51746d350f7cf63642a070fb5318#extracted.

